# Comparison between rapid and mixed maxillary expansion through an assessment of dento-skeletal effects on posteroanterior cephalometry

**DOI:** 10.1186/s40510-014-0046-9

**Published:** 2014-07-18

**Authors:** Letizia Perillo, Alfredo De Rosa, Francesco Iaselli, Fabrizia d’Apuzzo, Vincenzo Grassia, Salvatore Cappabianca

**Affiliations:** Department of Orthodontics, Second University of Naples, Naples, Italy; Department of Diagnostic Imaging, Second University of Naples, Via Luigi De Crecchio 6, 80138 Napoli, Italy

## Abstract

**Background:**

The aim of this study is to compare the dento-skeletal effects of rapid maxillary expansion (RME) and mixed maxillary expansion (MME), assessed on posteroanterior (PA) cephalograms.

**Methods:**

Treatment groups consisted of 42 patients; mean age in RME group (*n* = 21,13 female and 8 male subjects) was 8.8 years ± 1.37 at T0 and 9.6 years ± 1.45 at T1 and mean age in MME group (*n* = 21, 12 female and 9 male patients) was 8.9 years ± 2.34 at T0 and 10.5 years ± 2.08 at T1. Seventeen bilateral anatomic landmarks, 16 linear (12 skeletal and 4 dental) and 4 angular measurements were assessed for each patient at T0 and T1. Data from the two groups were compared using independent sample *t* test (*p* < 0.05).

**Results:**

At T0, the groups were similar for all examined variables (*p* > 0.05). Significant and equal increase of lateronasal and maxillary and upper and lower molar widths (*p* < 0. 01) occurred in both groups at T1. Significant but different increases were observed for maxillary incisal, upper left first molar-lateroorbitale, and maxillary first molar angles (*p* < 0.001 vs. *p* < 0.05). Significant increases were reported for upper inter-incisal width apex (*p* < 0.001) and upper right first molar-lateroorbitale angle (*p* < 0.05) only in the RME group. At T1, differences in maxillary incisal angle (*p* < 0.05), upper left first molar-lateroorbitale, and maxillary first molar angles (*p* < 0.001) were noted.

**Conclusions:**

RME and MME were both effective to increase skeletal transverse dimensions by opening mid-palatal suture in growing patients, while MME was associated with minor dental side effects than RME.

## Background

Maxillary expansion is widely used in growing patients in order to eliminate a transverse discrepancy between the dental arches due to maxillary constriction [1–4]. Treatment-induced widening of the maxilla leads to the correction of posterior crossbites [5–8], to the coordination of the maxillary and mandibular dental arches [9–13] and to gain in arch perimeter in patients with tooth size/arch size discrepancies [4,9].

Over the years, many methods have been used to expand the constricted maxilla, through rapid [3,4,10,14,15], semirapid [16], and slow expansion [5,7,17,18] based on the common aim for minimal dental and maximum skeletal effects [10].

Classical studies by Krebs [12] and Skieller [19] and the more recent by Akkaya et al. [7] affirmed that rapid maxillary expansion appliances showed the best examples of true orthopedics in that changes are produced primarily in the underlying structures and therefore are found to be more stable [8,20,21].

However, clinical and histological studies have shown that microtrauma of the temporomandibular joint, microfractures at the mid-palatal suture, external root resorption, and dental tipping are observed in rapid maxillary expansion treatment [7,12,19,22–25].

To eliminate these disadvantages and achieve a more physiological tissue reaction, slow maxillary expansion became more popular [7,26,27] although skeletal effects were less evident [7,12,19].

Finally, it should be considered that the high forces generated by rapid maxillary expansion (RME) and the rapid displacement or deformation of the facial bones would result in a marked amount of relapse in the long-term, whereas relatively slower expansion of the maxilla would probably produce less resistance in the nasal-maxillary complex [16].

These findings led Işeri and Ozsoy [16] to propose a protocol, named semi-rapid maxillary expansion (SRME) with RME followed by slow maxillary expansion, immediately after the separation of the mid-palatal suture. The schedule was two turns each day for the first 5 to 6 days, and three turns each week, the remainder of the RME treatment.

Because of a week of RME expansion may still have some side effects, we proposed a new protocol, called mixed maxillary expansion, which is able to separate the two maxillary halves at the first appointment so that the expansion forces were completely applied to the maxillary bone. The hypothesis was that this protocol might allow major skeletal and minor dental effects.

The aim of this study was to compare the transverse dento-skeletal effects in patients treated with RME and mixed maxillary expansion (MME) using posteroanterior (PA) cephalometric radiographs.

## Methods

We performed a retrospective study on 42 patients treated with maxillary expansion from October 2010 to March 2012.

Inclusion criteria were uni- or bilateral posterior crossbite and/or variable degree of tooth crowding, fully erupted upper permanent molars, mixed dentition, stage 1 or 2 of the cervical vertebral maturation (CVM), treatment performed with rapid or mixed maxillary expansion, dental casts, high-quality latero-lateral, postero-anterior, and occlusal radiograph at two time periods, pre-expansion (T0), and post-expansion (T1).

Patients with cranio-facial anomalies, severe periodontal disease, dental trauma or anomalies, and previous orthodontic treatment were excluded.

Two groups of patients, who met the inclusion criteria, were selected from the files of our department, 21 treated with rapid maxillary expansion and 21 with mixed maxillary expansion. We used a two-band palatal Hyrax-type expander applied in mixed dentition and bonded to the first upper molars and first deciduous molars or first bicuspids.

### Activation

In both groups, the activation started soon after the appliance was cemented and ended when overcorrection was achieved with the palatal cusps of the upper molars riding up on the buccal cusps of the lowers. The activation schedule of both protocols was given in Table 1.

**Table 1:** **RME and MME activation and retention procedure**

	**Expansion**				**Retention**	**Total treatment duration**
	**Phases**	**Turns**	**Duration**	**Controls**		
RME group	One, rapid	2/day	1 to 3 weeks	4/months	8 months ± 2	1.2 year ± 0.3
MME	First, very rapid	4-2-1/day	1 h	2/months	8 months ± 2	1.3 year ± 0.2
	Second, slow	2/week	4 to 6 months			

### RME group activation

In RME group, the operator began activation at the chair turning the expansion screw with two turns (0.25 mm per turn), then parents were instructed to continue the activation at home with two turns per day.

During the expansion phase, lasted from 1 to 3 weeks, depending on the degree of maxillary constriction and/or tooth crowding, patients were monitored once a week.

### MME group activation

In MME group, the activation was performed in two phases, the first very rapid and the second slow. The first, started at the chair, included three steps with four, two, and one turn (0.25 mm per turn), respectively. The three steps were performed in the same visit until the suture was opened. The decrease in tenderness on the bonded teeth and/or tenderness in the sutural area may indicate that maxillary halves have already been separated. Thus, an occlusal radiograph confirmed the successful separation, before and after the maxillary expansion. In the event of increased palatal suture resistance, a fourth step with two additional turns may be applied.

After opening the suture, the second expansion phase began and parents were instructed to continue the activation at home with one turn every 3 days.

During the expansion phase, lasted from 4 to 6 months, depending on the degree of maxillary constriction and/or of tooth crowding, patients were monitored once every 2 weeks.

### Retention phase

After the expansion phase, both groups had the Hyrax device removed to lock the screw with cold acrylic and then re-cemented so that it could be used as a retainer. The retention phase lasted on average 8 months. The retention schedule of both protocols was given in Table 1.

### Cephalometric analysis

PA cephalograms were hand-traced with a 0.5-mm lead on a 0.003-mm matte acetate tracing paper.

All tracings were performed by one investigator and verified a week later.

**Figure 1:**
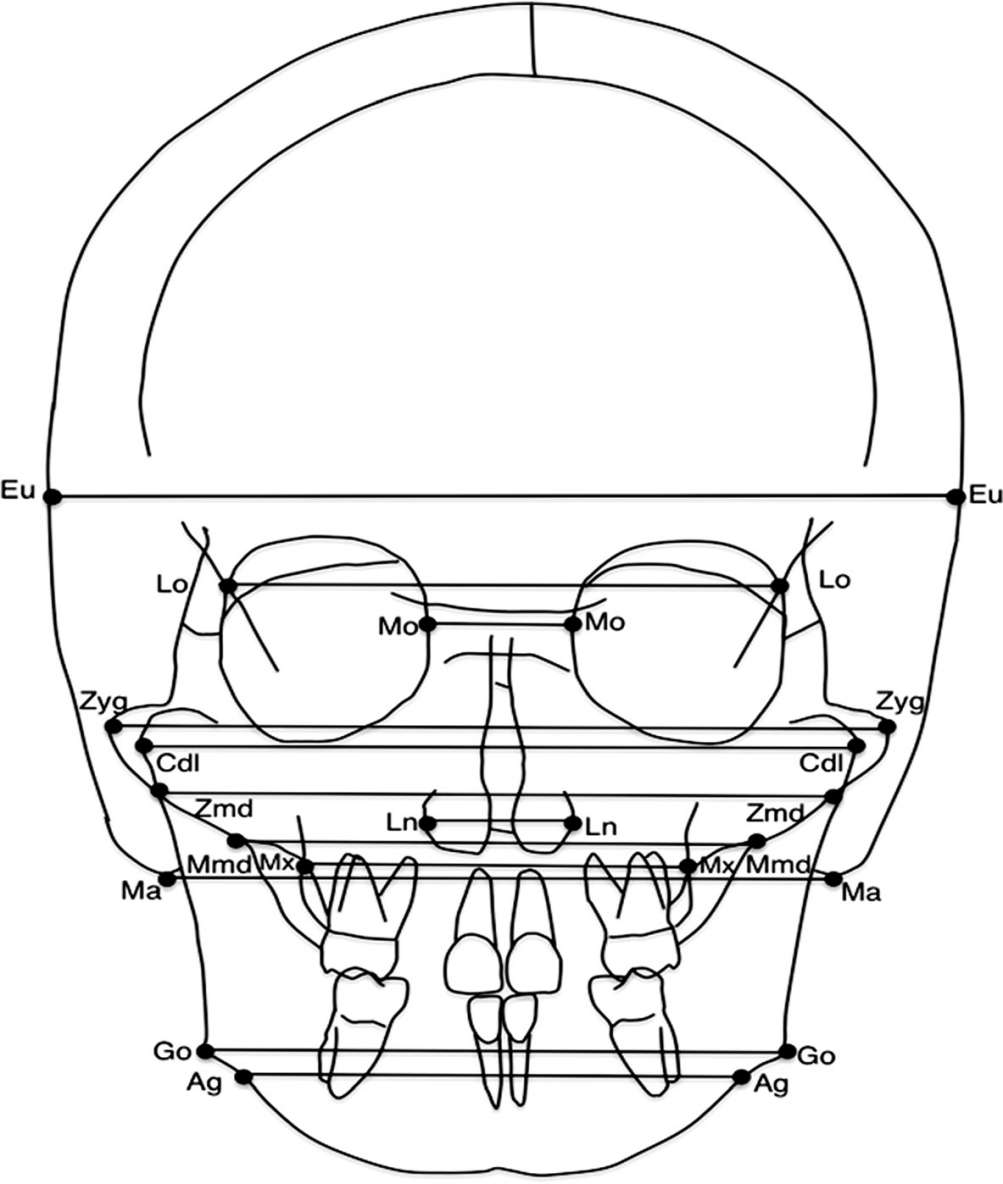
**Skeletal landmarks and measurements.**

**Figure 2:**
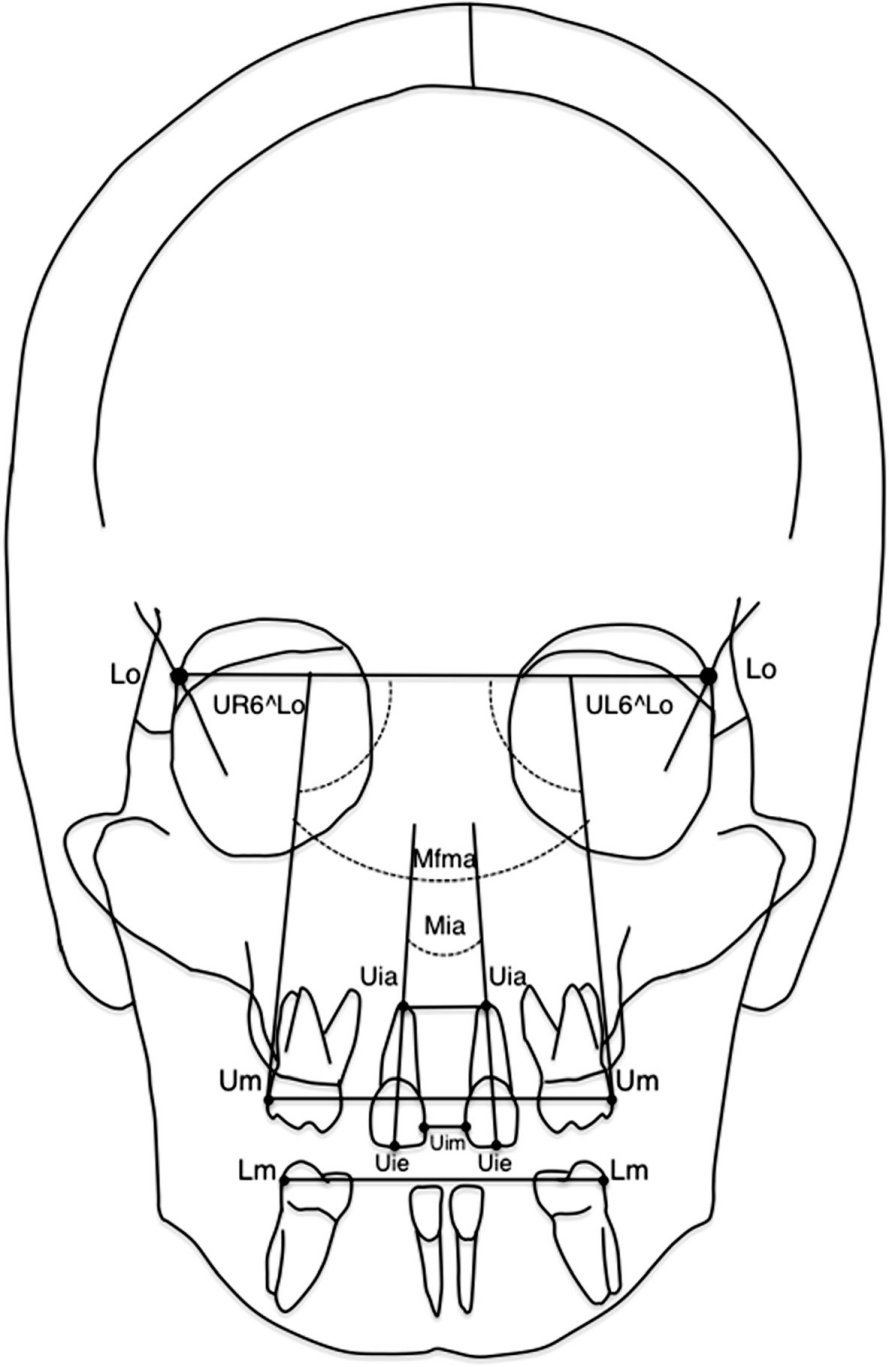
**Dental landmarks and measurements.**

Seventeen bilateral anatomic landmarks, 16 linear (12 skeletal and 4 dental), and 4 angular measurements were derived for each patient at T0 and T1. Definitions of dental and skeletal landmarks with linear and angular measurements were reported in Figures 1 and 2 and Tables 2 and 3.

**Table 2:** **Definition of skeletal landmarks and linear measurements**

**Skeletal landmarks**	**Linear measurements**
1. Euryon (Eu) - the most lateral point of the cranial vault	1. Euryon width
2. Lateroorbitale (Lo) - the intersection of the lateral wall of the orbit and the greater wing of the sphenoid (the oblique line)	2. Lateroorbitale width
3. Medioorbitale (Mo) - the most medial point of the orbital orifice	3. Medioorbitale width
4. Zygomatic (Zyg) - the most lateral point of the zygomatic arch	4. Bizygomatic width
5. Condylar lateral (Cdl) - the point located at the lateral pole of the condylar head.	5. Condylar width
6. Zygomandibulare (Zmd) - the intersection between the lower margin of the zygomatic bone and the lateral contour of the mandibular ramus	6. Zygomandibulare width
7. Lateronasal (Ln) - the most lateral point of the nasal cavity	7. Lateronasal width
8. Maxillomandibulare (Mmd) - the intersection between the lower margin of the maxilla and the medial contour of the mandibular ramus	8. Maxillomandibulare width
9. Maxillare (Mx) - the point located at the depth of the concavity of the lateral maxillary contour, at the junction of the maxilla and the zygomatic buttress	9. Maxillary width
10. Mastoid (Ma) - the lowest point of the mastoid process	10. Mastoid width
11. Gonion (Go) - the point located at the gonial angle of the mandible	11. Bigonial width
12. Antegonion (Ag) - the point located at the antegonial notch	12. Antegonial width

**Table 3:** **Definition of dental landmarks and linear and angular measurements**

**Dental landmarks**	**Linear and angular measurements**
1. Upper molar (Um) - the most prominent lateral point on the buccal surface of the upper first molar	1. Upper inter-molar width
2. Lower molar (Lm) - the most prominent lateral point on the buccal surface of the lower first molar	2. Lower inter-molar width
3. Upper incisor mesial (Uim) - the most mesial point of the upper central incisor crown	3. Upper inter-incisal width-mesial
4. Upper incisor apex (Uia) - the tip of the root apex of the upper central incisor	4. Upper inter-incisal width-apex
5. Upper incisor edge (Uie) - the point located on the incisal edge of the upper central incisor, centered mediolaterally	5. Maxillary incisal angle (Mia) - the angle between the major axe of upper central incisors (Uia-Uie).
	6. Upper right first molar-laterorbitale width (UR6^Lo) - the angle between the upper right first molar tangent and laterorbitale width.
	7. Upper left first molar-laterorbitale width (UL6^Lo) - the angle between the upper left first molar tangent and the laterorbitale width
	8. Maxillary first molar angle (Mfma) - the angle between the tangent of upper first molars

### Statistical analysis

Descriptive statistics were performed on cephalometric measurements at T0 and T1 for the RME and MME groups.

The following statistical comparisons were performed:

- Comparison of starting forms: RME group vs. MME group at T0
- Treatment effects: RME group T1–T0
- Treatment effects: MME group T1–T0
- Comparison of final forms: RME group vs. MME group at T1

Groups were compared using independent sample *t* test. Significance was set at 0.05 for all statistical analyses.

### Error study

To analyze the error of the method, the same examiner retraced 20 randomly selected PA cephalograms. A combined error of landmark location, tracing, and measurement was determined. The error standard deviation for each dimension was calculated by the Dahlberg's formula derived from Hald.

## Results

The main characteristics of the samples were summarized in Table 4. RME and MME groups were matched for number, sex, chronological age, and CVM stage. The RME group (*n* = 21) consisted of 13 girls and 8 boys with the mean age of 8.8 years ± 1.37 at T0 and 9.6 years ± 1.45 at T1. The MME group (*n* = 21) consisted of 12 girls and 9 boys with a mean age of 8.9 years ± 2.34 at T0 and 10.5 years ± 2.08 at T1. The CVM stage ranged from CS1 to 2 at T0 and from CS2 to 3 at T1. The mean value of the method error was 0.5 mm ± 0.2 mm.

**Table 4:** **Characteristics of RME and MME groups**

		**Male**	**Female**	**Mean age ± SD (years)**		**CVM stage**	
				**T0**	**T1**	**T0**	**T1**
RME group	21	8	13	8.8 ± 1.37	9.6 ± 1.45	CS1-CS2	CS2-CS3
MME group	21	9	12	8.9 ± 2.34	10.5 ± 2.08	CS1-CS2	CS2-CS3

Descriptive statistics for values and changes of the skeletal and dental measurements with comparisons were reported in Tables 5 and 6. Before treatment (T0), the groups were similar for all skeletal and dental variables examined (*p* > 0.05). The evaluation of the changes after RME and MME (T0–T1) showed significant and equal increase of lateronasal (*p* < 0.001) and maxillary (*p* < 0.001) and upper (*p* < 0.001) and lower (*p* < 0. 01) molar widths. Significant but different increases were observed for maxillary incisal (*p* < 0.001 vs. *p* < 0.05), upper left first molar-lateroorbitale (*p* < 0.001 vs. *p* < 0.05), and maxillary first molar (*p* < 0.001 vs. *p* < 0.05) angles. Significant increases were reported for upper inter-incisal width apex (*p* < 0.001) and upper right first molar-lateroorbitale angle (*p* < 0.05), only the RME group. Differences for other skeletal and dental measurements were not significant. After treatment (T1), groups showed the following differences, maxillary incisal (*p* < 0.05), upper left first molar-lateroorbitale (*p* < 0.001), and maxillary first molar (*p* < 0.001) angles.

**Table 5:** **Descriptive statistic: skeletal measures**

	**Initial**					**Change after treatment**				**Final**				
	**RME group**		**MME group**			**RME group**		**MME group**		**RME group T0**		**MME**		
	**T0**		**T0**			**T1-T0**		**T1-T0**		**T1**		**T1**		
**Skeletal measure (mm)**	**Mean**	**SD**	**Mean**	**SD**	***p*** **value**	**T1**	***p*** **value**	**Mean**	***p*** **value**	**Mean**	**SD**	**Mean**	**SD**	***p*** **value**
eu-eu	146.26	5.16	148.10	4.32	ns	0.72	ns	0.71	ns	146.98	5.22	148.81	4.13	ns
lo-lo	91.52	4.18	90.95	7.03	ns	1.53	ns	2.24	ns	93.05	4.33	93.19	6.86	ns
mo-mo	22.90	2.07	23.57	1.68	ns	0.74	ns	1.01	ns	23.64	1.91	24.58	1.79	ns
zyg-zyg	120.88	4.76	122.64	4.33	ns	2.50	ns	2.91	ns	123.38	4.84	125.55	5.51	ns
cdl-cdl	114.76	4.72	115.10	3.45	ns	1.81	ns	2.21	ns	116.57	4.55	117.31	3.62	ns
zmd-zmd	106.05	6.68	107.40	4.71	ns	1.71	ns	1.58	ns	107.76	6.40	108.98	4.76	ns
ln-ln	26.36	1.89	26.67	2.04	ns	2.85	***	2.97	***	29.21	1.61	29.64	2.64	ns
mmd-mmd	78.98	4.23	80.55	2.84	ns	2.16	ns	1.71	ns	81.14	3.75	82.26	2.77	ns
mx-mx	59.79	2.59	60.26	4.79	ns	6.07	***	6.57	***	65.86	3.12	66.83	5.76	ns
ma-ma	108.31	6.11	109.50	6.01	ns	1.31	ns	3.24	ns	109.62	5.03	112.74	7.51	ns
go-go	87.57	4.20	89.48	4.78	ns	1.45	ns	1.54	ns	89.02	4.65	91.02	4.65	ns
ag-ag	81.60	3.44	81.74	4.77	ns	1.90	ns	2.47	ns	83.50	3.23	84.21	5.04	ns

*p < 0.05; **p < 0.01; ***p < 0.001; ns, not significant.

**Table 6:** **Descriptive statistic: dental measures**

	**Initial**					**Change after treatment**				**Final**				
	**RME group**		**MME group**			**RME group**		**MME group**		**RME group**		**MME group**		
	**T0**		**T0**			**T1-T0**		**T1-T0**		**T1**		**T1**		
**Dental measure (mm/°)**	**Mean**	**SD**	**Mean**	**SD**	***p*** **value**	**Mean**	***p*** **value**	**Mean**	***p*** **value**	**Mean**	**SD**	**Mean**	**SD**	***p*** **value**
um-um	57.07	2.49	56.29	3.51	ns	9.17	***	10.21	***	66.24	3.54	66.50	5.53	ns
lm-lm	56.52	2.78	57.10	3.56	ns	2.67	**	3.54	**	59.19	3.32	60.64	3.87	ns
Mfma	7.64°	2.01	6.83°	1.95	ns	4.15°	***	1.59°	*	11.79°	1.90	8.42°	1.88	***
UR6^Lo	94.29°	1.74	94.17°	2.07	ns	1.95°	*	1.26°	ns	96.24°	2.87	95.43°	2.77	ns
UL6^Lo	93.40°	1.85	92.86°	1.92	ns	2.89°	***	1.26°	*	96.29°	1.57	94.12°	1.64	***
uia-uia	7.07	1.38	7.31	1.89	ns	3.03	***	1.40	ns	10.10	2.14	8.71	2.59	ns
uim-uim	1.24	1.02	0.98	1.21	ns	0.59	ns	−0.29	ns	1.83	3.20	0.69	0.95	ns
Mia	5.69°	6.33	6.07°	6.84	ns	−9.26°	***	−4.78°	*	−3.57°	7.77	1.29°	7.69	*

*p < 0.05; **p < 0.01; ***p < 0.001; ns, not significant.

## Discussion

During expansion, forces exerted by the expander separate the maxillary halves resulting in the opening of the mid-palatal suture.

The separation of the maxilla halves occurs through their lateral rotation with the center located on the fronto-nasal suture or on the spheno-occipital synchondrosis (Braun et al. [28]).

Upon new bone formation in the opened suture, the basal bone width is increased.

The early clinical sign of this orthopedic effect is the appearance of a diastema between the upper central incisors (da Silva et al. [3]), implying a decreased opacity between the two halves of the maxillary bones on occlusal radiographs [10].

The buccal tipping of the posterior teeth is one of the most important side effects of the maxillary expansion.

The working hypothesis was that MME separates the two maxillary halves at the first appointment so that the forces during the expansion are completely applied to the maxillary bone with major skeletal and minor dental effects.

The purpose of this retrospective study was to compare the transverse dento-skeletal effects in patients treated with RME and MME using PA cephalometric radiographs.

The method error was low, showing high reliability of location, tracing, and measurements, confirming that the use of PA cephalograms is still a useful method to assess transverse dento-skeletal changes.

### Comparison of starting forms: RME group vs. MME group at T0

At baseline, RME and MME showed similar skeletal and dental variables, so they were comparable.

### Comparison of treatment effects: T1–T0 changes in RME group vs. T1–T0 changes in MME group

After expansion, we observed in the RME and MME groups similar significant skeletal increases for lateronasal (ln-ln) and maxillary (mx-mx) widths.

Significant dental increases were observed in the two groups for upper molar (um-um) and lower molar (lm-lm) widths, maxillary incisal angle (Mia), maxillary first molar angle (Mfma), and upper left first molar-lateroorbitale angle (UL6^Lo) increases, whereas upper inter-incisal width apex (Uia-Uia) and upper right first molar-lateroorbitale angle (UR6^Lo) increased significantly only in the RME group (Tables 5 and 6).

The average increase in the nasal cavity (ln-ln) in the RME and MME groups was 2.85 and 2.97 mm, respectively. These values were greater than the mean increase found by Krebs (1.4 mm) [12] and Wertz (1.9 mm) [13]. Probably, this was related to the age of patients, 8.8 to 10.5 years old, in our sample, vs. 8 to 19 years old of Krebs' sample [12].

In both experimental groups, significant increase in the maxillary transverse widths (mx-mx) of about 6.07 mm in the RME group and 6.57 mm in the MME group was obtained. The amount of maxillary expansion did not differ significantly between the two groups and was greater than that shown in the literature (Martina et al. [6]).

Previous studies about RME conducted by Cross and McDonald [15] showed a maxillary width increase of about 1.11 mm in 13.4-year-old patients, whereas da Silva [3] obtained an increase of about 2.81 mm in 5 to 11-year-old patients.

Since our patients were younger than those patients in Cross and McDonald's study (8.8 to 10.5 years old vs. 13.4 years), with a less skeletal maturity (CS1-CS2), sutures may have exhibited a lower resistance to expansion forces leading to a greater skeletal expansion.

Previous studies about SME conducted by Defraia et al. [18] in a sample of 6.2-year-old patients treated with removable appliance showed an increase in the maxillary width of about 4.48 mm, whereas Işeri et al. [16] in sample of 14.57-year-old patients treated with a rigid acrylic maxillary expander for 4.08 months found an increase of about 2.47 mm. This can be due to the different skeletal maturity of the two samples according to Baccetti et al. [9].

Both expansion modalities produced increases in the upper molar transverse widths (um-um) of about 9.17 mm in the RME group and 10.22 mm in the MME group, with no significant difference between the groups. These results are in agreement with previous data concerning RME by Lagravere et al. [20,29], which reported an average transverse increase of 6.7 mm, though our findings are greater as a result of greater maxillary width increase.

Both expansion modalities produced increases in the lower molar transverse widths (lm-lm) of about 2.67 mm in the RME group and 3.54 mm in the MME group, with no significant difference between the groups.

This finding would appear to support previous findings that uprighting of lower molars can occur (Gryson [30,31]; Sandstrom et al. [32]).

Comparison between the expansion modalities showed increases in the molar tipping measured (Mfma) of about 4.15° in the RME group and 1.59° in the MME group, with significant difference between the groups.

The literature confirms that the increase in maxillary width is attained through a separation of two maxillary processes (orthopedic effect) and buccal tipping of the teeth and alveolar processes (orthodontic effect) (da Silva et al. [3]).

Our results underscored major orthodontic effect in the RME group than in MME group as a side effect of this expansion modality.

A systematic review conducted by Lione et al. [33] concluded that heavy forces produce an increased buccal inclination of anchored teeth at the end of expansion.

Furthermore, in both groups, asymmetrical tipping of the respective anchored teeth was observed more often than not. This result is in agreement with previous data concerning RME by Asanza et al. [14], who reported that most patients demonstrated a wide variation of angular change from one side to the other. In our study, in the RME group, both UR6^Lo and UL6^Lo increased significantly, 2.89° and 1.95°, respectively, whereas in the MME group only UL6^Lo increased of 1.26°.

The behavior of the anterior segments of the maxilla was appraised by the upper inter-incisal width-mesial (Uim-Uim), upper inter-incisal width-apex (Uia-Uia), and maxillary incisal angle (Mia).

In the RME group, the root apices moved further laterally than the crowns, 3.03 mm (Uia-Uia) and 0.59 mm (Uim-Uim), respectively.

Haas explained this fact in 1961, thanks to the transeptal fibers tending to keep the proximity of the central incisor crowns [10].

Incisors' movement decreased significantly the angle formed by long axis of these teeth (Mia), of about 9.26°.

Similarly, in the MME group, the upper central incisors were laterally separated, the apices more than the crowns. More precisely, the apices moved slight laterally with a mean value of 1.4 mm (Uia-Uia), whereas the crowns tipped toward the midline, determining a decreasing in upper inter-incisal width-mesial (Uim-Uim) of 0.29 mm.

Maxillary incisal angle (Mia) in the MME group was significantly decreased of a mean value of 4.78°.

To our knowledge, there are only a few studies evaluating upper inter-incisal width-mesial (Uim-Uim), upper inter-incisal width-apex (Uia-Uia), and maxillary incisal angle (Mia). Previous long-term study conducted by Cameron et al. [4] about RME showed results closer to our MME outcomes than RME, probably as a result of the adaptation process that occurs during slow phase.

### Comparison of final forms: RME group vs. MME group at T1

At T1, the two groups overlapped for skeletal increments, whereas three dental variables were significantly different (Tables 5 and 6).

In the RME group, maxillary incisal angle (Mia) was negative, −3.57°, in spite of the value in MME group, 1.29°. This result showed the possibility of adaptation process in circum-maxillary structure during the slow phase in MME that does not occur in a protocol with only rapid approach (Işeri and Ozsoy [16]).

Maxillary first molar angle (Mfma) was significantly greater in the RME (11.79° vs. 8.42°). Since maxillary widths were not significantly different between groups, the greater value of maxillary first molar angle in the RME group was associated with buccal tipping of the upper molars.

Moreover, the asymmetrical pattern of buccal tipping in the RME group, already demonstrated by comparison of treatment effects, was confirmed by comparison of upper left first molar-lateroorbitale angle (UL6^Lo) at T1, which reported a greater value in the RME group (96.29° vs. 94.12°).

## Conclusions

RME and MME were both effective to increase skeletal transverse dimensions by opening mid-palatal suture in growing patients. Thus, results did not confirm the hypothesis that MME may allow major skeletal effects.

Statistically significant differences in dental measurements effects were found. Both expansion modalities resulted in tipping of the posterior teeth, greater on one side, and reduction of the maxillary incisor angle. These side effects were significantly greater in RME group. These outcomes suggested that MME was associated with the same skeletal effects and minor dental side effects than RME.
